# Redeeming qualities: exploring factors that affect women’s use of reproductive health vouchers in Cambodia

**DOI:** 10.1186/1472-698X-13-13

**Published:** 2013-02-26

**Authors:** Carinne D Brody, Julie Freccero, Claire D Brindis, Ben Bellows

**Affiliations:** 1 School of Public Health, University of California, Berkeley, 50 University Hall, #7360, 94720, Berkeley, CA, USA; 2 Public Health Program, Touro University, California, 1310 Club Drive, 94592, Vallejo, CA, USA; 3 Philip R. Lee Institute for Health Policy Studies & Bixby Center for Global Reproductive Health, University of California, San Francisco, 3333 California Street, Suite 335, Box 0744, 94143-0744, San Francisco, CA, USA; 4 Population Council – Kenya, Reproductive Health Program, Ralph Bunche Road, P.O. Box 17643, Nairobi, Kenya

## Abstract

**Background:**

One approach to delivering healthcare in developing countries is through voucher programs, where vouchers are distributed to a specific population for free or subsidized health care. Recent evaluations suggest that vouchers have the potential to extend coverage of priority health services to the poor in developing countries. In Cambodia, a reproductive health voucher program was implemented in January 2011. This study aims to explore women’s early experiences accessing health services with their vouchers at accredited clinics.

**Methods:**

This qualitative exploratory study used focus group methodology to gather information from five groups of older (>25 years) and four groups of younger (18–25 years) women who were eligible for the voucher program in three rural provinces. Focus groups were digitally recorded, transcribed and translated from Khmer into English. Data analysis was an iterative process, which comprised of open coding to find commonalities that reflected categories or themes and axial coding to relate initial themes to each other. Next, a basic framework for analysis was formed by integrating the themes into the framework.

**Results:**

Two overarching themes were identified in the data: 1) factors that facilitate voucher use and 2) factors that inhibit voucher use. Within each of these themes, three subthemes were identified: 1) pre-existing factors, 2) distribution factors, and 3) redemption factors. Overall, women expressed positive feelings towards the voucher program, while several areas for program improvement were identified including the importance of addressing pre-existing demand-side barriers to using reproductive health services, the need for more comprehensive counselling during voucher distribution, and the persistent cost of unofficial payments expected by midwives after delivery irrespective of voucher use.

**Conclusions:**

Early information from program beneficiaries can lead to timely and responsive changes that can help to maximize program success. This study highlights the importance of tailoring voucher programs to specific community needs, a strategy that can lead to better program uptake.

## Background

Within Asia, Cambodia has some of the poorest maternal health indicators. Following years of war, genocide, and occupation, the country has struggled to rebuild its health care system. The maternal mortality ratio is estimated at 266 deaths per 100,000 live births -- the second highest in the region [1]. The low prevalence of contraception use for spacing and limiting births, the low use of skilled attendance at birth, and the lack of emergency obstetric care when complications arise are seen as the leading contributors to these high rates of death [2]. Another contributing factor pertains to women’s roles in the society. According to a 2004 population survey, about 40% of rural women in Cambodia are illiterate [3]. The total fertility rate in rural communities is approximately 4 children per women, approximately one child more per woman than in the urban areas of Cambodia [4]. The total fertility rate of the poorest wealth quintile is 4.9 [4].

In Cambodia, the population relies upon a mixture of public facilities, private providers, private drug sellers and traditional healers. The quality of the public system has suffered from a lack of resources; complaints of long waits and staff shortages reflect the funding constraints within the public system. But the poorest continue to rely on public services because they have been priced out of most private healthcare options.

User fees were introduced in public health facilities in Cambodia in 1997 with the goal of injecting funds into the health system, which in turn, would be expected to enhance the quality of services. Because of government subsidies and donor support, user fees were set lower than private healthcare fees. But studies from around the world have shown that user fees have a strong negative effect on the use of healthcare services by the poorest [5,6]. Ten years later, more than two thirds of total health expenditures were derived from direct out-of-pocket payments (USD 35 per capita, 2007), contributing to unmanageable healthcare-related debt, hitting the poor the hardest [7]. The increasing need for developing fiscal safety nets for the poor and vulnerable was clear.

In an effort to extend the reach of public health care coverage to the poor, especially in the domain of child and maternal health, the Cambodian government developed a National Social Protection Strategy for the Poor and Vulnerable in 2000. Part of this strategy includes the Health Equity Fund, which is designed to reduce financial barriers to accessing health services at the provincial hospital level. Evidence from several studies suggests that these funds have successfully reduced out-of-pocket payments and health care-related debts [8]. In 2007, the government set out to work with various foreign donors to find a complementary strategy to the existing Health Equity Fund that would extend to the primary care level. Several pilot programs were launched to ascertain if voucher schemes might be the answer. Evidence from one pilot program that was tested in 2008 suggested that a voucher scheme contributed to increasing the number of deliveries in public health facilities [8].

In 2011, the German Development Bank, *Kreditanstalt für Wiederaufbau*, partnered with the Cambodian Ministry of Health to roll out a larger scale reproductive and maternal health voucher program. With a voucher, poor women can overcome some of the barriers to accessing family planning and safe delivery services including out-of-pocket and transportation costs. The Cambodia Vouchers for Reproductive Health Project is managed by Action for Health, a Cambodia-based organization, and is being evaluated by the Population Council. The program gives poor women a voucher to access quality services from pre-approved providers who are reimbursed for providing services to the voucher client. This is one of two health care subsidy programs that have been implemented in the area; Marie Stopes International launched a voucher program for reproductive health services in 2010.

Within this system, subsidized vouchers are distributed to poor households for a specific health good or service, such as maternal care or family planning, along with information about the benefits of birth spacing and limiting as well as how and where they might obtain the services, thus, eliminating traditional barriers to care. These services are targeted explicitly because they are know to be evidence-based and cost-effective.

Patients redeem these vouchers at accredited facilities, which have undergone a quality assessment in order to demonstrate their ability to provide the types of standards of care required, and have signed a contract with a third party agency, often known as voucher management agencies (VMA). A third party VMA (i.e., a non-governmental organization or private company) processes claims from facilities for each voucher patient visit and delivers reimbursement funds provided by donors to either government ministries or directly to the third party agency.

In return for steady, reliable income for the increased utilization of services by clientele, clinics are incentivized to maintain high quality services in order to satisfy patient demand, because otherwise, these same patients would be eligible to receive care through other settings. In sum, by targeting specific populations, increasing access and utilization, and enhancing quality and efficiency, it is anticipated that voucher programs will significantly improve the health of populations.

### Cambodia program design

The Vouchers for Reproductive Health Project was launched in partnership with the Ministry of Health in early 2011 in three provinces in Cambodia: Kampong Thom, Prey Veng and Kampot. Within this system, subsidized vouchers are distributed to poor households. Patients receive vouchers for maternity care, family planning and abortion, along with counseling about the types of services offered. As is true of other voucher programs, the facilities are accredited and have signed up with a VMA, led by EPOS Consulting and implemented by Action for Health. Action for Health processes claims from facilities for each voucher patient visit and reimburses the Ministry of Health’s government clinics, as well as several non-profit Marie Stopes International clinics.

All providers at participating clinics were officially salaried employees, although, as will be explained in more detail later, unofficial payments to public health providers is a common practice. Apart from accrediting public clinics, the VMA was charged with hiring and training distributors and overseeing voucher distribution. Voucher distributors were trained and supervised by a voucher agent at each operational district and by an overall provincial coordinator. The Ministry of Health provided education sessions for distributors and supplied them with leaflets and other educational materials. Vouchers are offered for family planning counseling and services, prenatal care up to 4 visits, delivery services, postnatal care up to 6 weeks postpartum, abortion services. In addition, a transportation stipend based on kilometers travelled is provided in cash to women at facilities.

Distributors were positioned throughout 9 operational districts within the 3 provinces: Kampong Thom (Stung, Kampong Thom and Baray Santuk); Prey Veng (Preasdach, Peareang and Kampong Trabek); Kampot (Angkor Chey, Kampong Trach and Chhouk). These three provinces are geographically diverse and represent one southern, coastal province (Kampot), one province just outside the capital city (Prey Veng), and one in the northern part of the country (Kampong Thom). Most clinics enrolled in the program in the three provinces were public health facilities. The private Marie Stopes clinics were only accessible to those participants from the Kampong Thom region.

Program eligibility for the voucher and, in turn, this evaluation, was pre-determined by the Ministry of Planning using a poverty grading scale to determine if a family falls under a pre-determined poverty line. If they were determined to be poor, they were given a numbered poor identification card with a photo of the family. This card gives households access to the Health Equity Fund, which covers all care at the district hospital. The voucher program, then, acts to extend the fund’s reach to the local health clinics. The poverty grading scale measured physical household characteristics, such as the type of roofing or flooring and family assets, such as ownership of a bicycle or amount of land. Distributors approached households that were identified as poor households. The total number of vouchers distributed by February 2012 was 3,523 for safe motherhood services and 15,631 for family planning [9].

The purpose of this study was to gather early information about women’s experiences with vouchers. Information gathered prior to the start of the program using hypothetical scenarios or behavioral intention questions have proven to produce inaccurate results [10]. Thus, this study aims to collect information about experiences early in the implementation of the initiative in order to ascertain whether program modifications are needed.

This study will explore women’s experiences with and perceptions about: 1) accessing health care services prior to the voucher program, and 2) redeeming their reproductive health vouchers for services at accredited facilities since the program started.

## Methods

### Design

This qualitative exploratory study used modified grounded theory and focus group methodology to gather information on the shared experiences of both older (>25 years) and younger (18–25 years) women who were eligible for the voucher program in the three program provinces. Grounded theory was chosen as the most appropriate method for this study because the research team was interested in uncovering and understanding the experiences and decision-making processes of Cambodian women around accessing healthcare with a voucher.

### Participants and recruitment

Ethical approval was sought and approved by both the University of California at Berkeley (Approval #: 2011-01-2722) and the Ministry of Health in Cambodia through the Population Council. Villages in the three provinces that had been visited by voucher distributors were randomly selected from the list of all participating villages and village chiefs were approached to participate. Once village chiefs accepted the invitation to participate, a date was set for the focus groups discussions. Village chiefs then informed program-eligible women in their villages to attend focus groups on the appointed day. Verbal informed consent was obtained from each focus group participant. Other than participants’ age, no other demographic data were collected.

### Data collection

Experienced facilitators from a Cambodian research institute who were oriented to this study led the focus groups using a structured focus group guide which was developed by the authors in collaboration with the Population Council. The focus group format allowed participants to both share individual experience and to exchange commonly held ideas and experiences. Each focus group consisted of 8–12 women and lasted a minimum of 2 hours and a maximum of 3 hours. All groups were held at the village chief’s house, which was an easily-accessible, safe, and familiar place to all participants.

Focus group guides included questions about women’s perceptions of their most pressing health needs, their experiences past accessing health care, their knowledge of the voucher program, and their experiences receiving and redeeming the voucher.

Focus groups were digitally recorded, transcribed verbatim and translated from Khmer into English. In addition, detailed memos were taken during the groups to record additional data, including non-verbal and environmental information, and these notes were used to de-brief with the research team immediately following each focus group. In addition, these notes supported translation and aided in analysis. Transcripts were uploaded into a qualitative data analysis software package, Nvivo version 9 software [11].

### Data analysis

Data analysis was an iterative process, which comprised of multiple forms of coding. Researchers first engaged in a process of open coding [12] to find commonalities that reflected categories or themes that we used to begin to develop broad themes. We then used axial coding [13] to relate initial themes to each other and form a basic framework for analysis. Once we had developed and tested a preliminary framework, we employed theoretical coding, which comprised of integrating the textual data into our emerging theoretical framework [13].

In order to obtain inter-rate reliability, two researchers generated initial codes for emerging themes independently. Any differences in codes, which were mostly differences in terminology, rather than differences in concepts, were resolved through consensus.

Through memos and de-briefing, the research team decided that we had reached theoretical saturation once we had conducted three focus groups in each province for a total of nine. Four of the groups were held with women 18–25 years old and five of the groups were held with women over 25 years old. Overall, a total of 81 women self-selected to participate in this study. In the following section, we describe the framework that emerged from the focus groups discussion in response to the structured questions, as well as specific illustrations of themes and sub-themes.

## Results

Six to ten women attended each focus group. A framework was developed iteratively during the data collection period and refined during data analysis reflecting the factors affecting voucher use that emerged from the focus groups and researcher discussions (Table 1).

**Table 1:** Factors that affect voucher use

Facilitating factors*		
**Pre-existing**	**Distribution**	**Redemption**
• Existing demand for family planning (9/9)	• Community-based distribution (6/9)	• Cost (free) (9/9)
• Knowledge of health risks (9/9)	• Support and facilitation by village chief (6/9)	• Transportation reimbursement (9/9)
• Positive experiences at health centers (9/9)	• Information about health services (3/9)	• Ease of use (9/9)
• Joint decision-making about family planning with partners (5/9)		• Perceived quality of life improvements (5/9)
• Personal decision-making about family planning (3/9)		
Inhibiting factors*		
**Pre-existing**	**Distribution**	**Redemption**
• Experiences with side effects of contraception (5/9)	• Confusion about voucher program logistics (4/9)	• Limited locations and services (3/9)
• Male partner resistance to family planning (3/9)	• Perceived poverty misclassification (3/9)	• Confusion with other programs (3/9)
• Informal costs or “tea money” (3/9)	• Missed distribution opportunities (3/9)	• Negative reactions from providers at hospitals (3/9)
• Preference for traditional care (3/9)		• Fear of hidden costs (2/9)

* Fractions in parentheses indicate the number of focus groups during which this category was discussed over the total number of focus groups.

Based on participants’ discussions about accessing services before and after the voucher program, two overarching themes were identified in the data: 1) facilitating factors and 2) inhibiting factors. Within each of these themes, three subthemes were identified: 1) pre-existing factors, 2) distribution factors, and 3) redemption factors. Multiple categories were developed under each sub-theme. These categories, subthemes and themes, which emerged within each area, are summarized in the table above and described with illustrative quotations from participants in the following sections.

## Overarching theme 1: factors that facilitate voucher use

### Sub-theme 1: pre-existing factors

#### Demand for family planning

Participants discussed at length the factors that facilitated their interest in seeking reproductive health services that pre-dated the voucher program. In all nine focus groups, women expressed having a desire to space or limit the number of children they had - mainly for economic reasons.

Birth spacing is very important to me. Having too many children means I don’t have time to earn money. I am busy taking care of the children and have no time to work (Kampot Province, Over 25 yr-olds).

#### Knowledge of risks

Other reasons for choosing to use reproductive health services included the knowledge of the health risks of closely-spaced births. Women in all nine focus groups said that they became weak when they had children too often. Women also recognized the importance of attending antenatal visits for iron supplements and check-ups and of going to the health center for delivery services after hearing about the women in their community who experienced complications or death during pregnancy and childbirth.

I use contraception so we don’t have any more children. Having them is not easy, it affects our health, and I had swelling last time. (Angkor Chey, Over 25 yr-olds).

During delivery, some women afraid for their situation. They think about what will happen to them, and it is not as they wish. Some women cannot deliver placenta after delivery, have vaginal bleeding and breathing problems and they need to get some injection and serum to gain their energy back. (Kampong Trabek, Under 25 yr-olds).

#### Positive experiences at health centers

In all nine focus groups, women reported that they were familiar with their closest public health center. Most participants expressed having had positive experiences seeking care there. Their local public health center was reported as being accessible to most women by bicycle or on foot and many women had the opinion that the cost for services was reasonable. Women reported having had positive interactions with medical staff and having been able to get advice about family illness, uncomfortable side effects of medications, and family planning options. Most women felt that the public health center offered an adequate level of service quality for these services.

I choose to go to the [public] health center because it is close and it is safe for both mother and child. The doctors are qualified and they are friendly. They take care of us (Kampong Thom Province, Over 25 yr-olds).

It’s easy and if we get sick or stomachache at night, we just call a nurse, we have their number and call them, they’ll arrive and wait for us at the health center (Kampong Trabek Province, Under 25 yr-olds).

#### Joint decision-making regarding contraception

Finally, women discussed their decision-making power in terms of who within their household makes reproductive healthcare decisions. In five of the nine focus groups, women expressed that they and their husbands made family planning decisions together.

If our family wants to have a child or wants to use contraception, we talk. Husband listens to wife and wife listens to husband. We listen to each other (Kampot Province, Over 25 yr-olds).

I have two children already and we discussed together. We are poor and having more children means we will become poorer and poorer so we decided to use family planning, birth spacing (Chuk 18–25 yr olds).

#### Personal decision-making regarding contraception

When asked about situations where there is a disagreement between husband and wife about fertility, responses varied. In three out of the nine focus groups, women reported that if they wanted the service and their husband disagreed, they would still go. Many of these women also expressed that if they had already “given many children to their husband,” they felt more comfortable going for services despite disagreement; this was reported by women in 2 focus groups and only by women over 25-years-old.

I make the decision. This is how I decide. When we have many children, the husband doesn’t care. They only go to work, but we are the ones at home (Kampong Thom Province, Over 25 yr-olds).

For me, even if my husband doesn’t let me go, I still go because it would be me who is experiencing the problems (Kampong Thom Province, Over 25 yr-olds).

I discuss with my husband but if my husband is not here, then I decide for myself (Angkor Chey, Over 25 yr olds).

### Sub-theme 2: distribution factors

#### Community-based distribution

At the time of the focus groups, most women had received their voucher through community-based distribution. Women reported that the organization delivered the vouchers to the village chiefs’ house or to their homes directly. This was discussed as a positive aspect of the program since they did not have to take the time or initiative to seek out the voucher on their own.

They distribute the cards throughout the commune, village, and our district. They distribute house by house. It is the village chief who visits and tells every house about the program. (Pearang Province, Over 25 yr-olds).

#### Support and facilitation by village chief

Women in five groups reported that program staff worked with the village chief to distribute vouchers to the homes of all families who were considered poor using the poverty grading scale mentioned earlier.

I got this voucher because the village chief went to my house and saw that we are poor; that is why he gave it to us (Chuk Province, Under 25 yr-olds).

#### Information about health services

During distribution visits, women in three out of the nine groups reported receiving reproductive health information, such as availability of contraception at the health center and the importance of birth spacing.

Staff came down to the village and they told us which contraception is available. We can use implant, tie the uterus, or take OK [brand of oral contraception], or injection. They have all kinds of contraception (Kampot Province, Over 25 yrs old).

There were no specific questions in the focus group guide asking about the quality or content of the counseling that is intended to happen during voucher distribution and few mentions were made of the quality of this educational component in the group discussions.

### Sub-theme 3: redemption factors

In each of the nine focus groups, there was at least one woman who reported she had redeemed her voucher. The women who had received but not redeemed their vouchers had either not yet had the opportunity to use them (not pregnant, not using contraception) or preferred to hear about other women’s experiences before trying it out themselves.

She hasn’t used her voucher because her husband wants three more children (Angkor Chey, Over 25 yr olds).

I don’t want to use it yet because it is not necessary (Angkor Chey, Over 25 yr olds).

#### Cost (free)

In nine out of nine focus groups, women agreed that the most attractive part of the voucher programs was the reduced cost of services. Women reported that being so poor made everything in their lives more difficult, including seeking services at the public health center despite what many women considered reasonable fees.

The advantages of this voucher are free of charge pregnancy checking, delivery and birth spacing. This voucher will help to eradicate poverty (Chuk Province, Under 25 yrs).

#### Transportation reimbursement

The transportation reimbursement provided by the voucher program was attractive to women in every group and provided additional encouragement for them to use the services that they wanted.

They said that we go with this voucher to get injection and they will not charge us and instead will give us some money for transportation to come home. They give us 5000 riel (approximately USD 1.25). Like [name of community member], she went there and they gave her 5000 riel as I heard (Kampot Province, Over 25 yrs old).

When you have the voucher, they have the motorbike taxi services, it is free (Angor Chey, Over 25 yr-olds).

#### Ease of use

Women who had use their voucher stated that they felt equally well received at the health center when they presented with the voucher as when the presented without it and that they had an easy time redeeming the voucher for services.

When we come with the voucher, we see the doctor, they inject serum [injectable contraceptives] immediately and they give back 7000 riels (approximately USD 1.75) (Prey Veng Province, Over 25 yrs old).

Women reported that community members who were eligible for the program would go to the health center more frequently now and that people who had never visited the health center before, perhaps because of cost, would now be able access these specific services.

When we have the voucher, we can go more often because we don’t have to spend the money (Kampong Thom Province, 18–25 yr-olds).

I think it is good because some people cannot afford to go to the health center, but with the voucher, it is free and those who have never gone before would start going (Kampong Thom Province, Over 25 yr-olds).

Before getting the voucher, I didn’t go to the doctor, not in two or three months. Now with the voucher, I go to the doctor more often and also get medicines. (Angor Chey, Over 25 yr-olds).

#### Perceived quality of life improvements

In five out of the nine focus groups, women who had used the voucher expressed that they had a better quality of life since the program began, including better physical health, mental ease, increased hope, and a feeling of security. The below quotes illustrate the various responses.

As soon as I had the voucher, I could go to tie my Fallopian tubes right away according to my dreams (Kampong Thom Province, Over 25 yr-olds).

I will tell my own experience. Before I didn’t care about birth spacing but after I got this voucher, I want to take care of my health and go to get birth spacing regularly like the doctor said (Chuk Province, 18–25 yr-old).

When we are home, we won’t get better. If we go to the doctors, they treat us and give us serum. It is easy. We can now hope to survive (Kampong Thom Province, 18–25 yrs old).

[Since having the voucher] it feels like having a mother at home, leaving money for us. No matter how short we are on money, the mother will always have it for us (Kampong Thom Province, Over 25 yr-olds).

## Overarching theme 2: factors that inhibit voucher use

### Sub-theme 1: pre-existing factors

#### Side effects of contraception

Fears about side affects are a barrier to using vouchers. While many women desired to limit or space the number of children that they have, it in five out of nine focus group women expressed that they shared common fears and concerns about the side effects of contraceptives that they had experienced or that they had heard about other women experiencing. These side effects included making them feel weak, making their breastmilk “hot”, giving them fevers, affecting their weight, inhibiting their ability to work in the fields, and causing irregular bleeding.

The doctor said that I have many children so I should use contraception. I had one injection but it didn’t work with my health. I had blood discharge every month so she said to take the pills instead. But I didn’t take them (Kampong Thom Province, Over 25 yr-olds).

#### Male resistance to family planning

Finally, some household decision-making patterns around health care seeking behavior were also inhibitive. As mentioned earlier, in five of the focus groups, women expressed feeling empowered to make family planning decisions either in collaboration with their husbands or on their own, while in three focus groups, women felt that if their husbands did not agree with their desire to use family planning, then they would not use these services in order to avoid conflict or other negative consequences. These sentiments were expressed by participants in focus groups held with women of both age groups.

If [husbands] want [more children] and we resist, then arguments will happen and trouble. So, we have to follow them (Kampot Province, Over 25 yr-olds).

If they want more children and we stand against them, they will walk away. They will stop giving us the money. They will leave us and the kids. They won’t support us financially anymore (Kampot Province, Over 25 yr-olds).

#### Informal costs

Even though some women felt that the cost of services was reasonable, other women felt that any additional cost burden was too much for them. A new issue emerged pertaining to costs that had not been discussed openly before. Participants, focus group facilitators, and program staff shared that delivering in a public health facility in these communities comes with an extra cost, which women referred to as “tea money”. Tea money is an unofficial payment of gratitude paid to a midwife that is considered by most to be required at public health centers and hospitals. Women reported that this payment was greater than what they would pay for a traditional midwife, who they may be able to pay in-kind, for example, with food or services such as sewing. Thus, having a voucher would not necessarily reduce the cost burden that many of the women already felt in using health center services.

In rural Cambodia, delivery fees for births attended by trained midwives or nurses cost five times as much as those attended by traditional birth attendants (TBA) ($27 vs. $6). These costs include “unofficial fees” that can be as much as $10 paid to the health providers at the time of delivery [14]. In addition, there are transportation, accommodation, food and other miscellaneous costs that are incurred when delivering in a facility [14]. In three out of nine focus groups, women reported that these unofficial costs have inhibited them from delivering in facilities and that the availability of vouchers did not totally eliminate the cost barriers they experienced.

If we have little money they are not happy and we have to pay tea money for thanks. We have to pay for the bed and tea money and if that is not enough, they just ask me to pay more. They will not let us leave the [public] hospital until they receive money (Kampot Province, Over 25 yr-olds).

Yes, we must have someone come home and get money for [the midwives] (Kampot Province, Over 25 yr-olds).

#### Preference for traditional care

Another factor that may inhibit women from using their vouchers for these services is their preference for traditional practices around childbirth. In three focus groups, women reported having had all their children at home, either by themselves or with a TBA, without experiencing any complications and saw no reason to change that practice. It was easier and cheaper than going to the health facility. Traditional birth attendants come directly to their homes. In addition, transferring to the health center during labor was uncomfortable.

I have never delivered in the hospital. All my three children were delivered at home. It was easy for me to deliver – the [TBA] came late and my child was already born (Kampot Province, Over 25 yr-olds).

[The traditional midwife] comes whenever we call her…The most important thing is that she comes to our house (Ko Koh Province, 18–25 yr-olds).

### Sub-theme 2: distribution

#### Confusion about voucher program logistics

The most commonly cited issue with the distribution process was that many women reported not understanding the program logistics. In four our of nine focus groups, women expressed not knowing where they could redeem their vouchers, where to get additional vouchers, or precisely which services were included. Many women reported relying on the village chief for information about the program. Although the village chief was consulted about the implementation of the project, individual women did not need to receive permission from the chief in order to get a voucher, thus, potentially missing an opportunity to hear more specific information about what the vouchers could offer them.

[If she wants a voucher] she should come to see the Village Chief or something…to help…I don’t know what else to say (Kampong Thom Province, Over 25 yr-olds).

I don’t know where they are available. I just know that the poor get them. Other than that, I don’t know where they come from or what they are for (Kampong Thom Province, 18–25 yr-olds).

#### Perceived poverty misclassification

In three out of nine focus groups, women reported feeling concerned about how the poverty classifications were made. Poverty classifications were made by voucher program staff who visited the village and went door-to-door assessing poverty levels based on a set grading scale prior to the start of the program. Several women felt that they or a community member had been misclassified.

*This house cannot get it. It is not considered poor. The roof is brick or metal. Unless you are living in a cottage then they won’t consider you poor. Like me, I don’t have a house, but I live with my mother so they don’t consider [me poor] as long as we have a house to live in* (*Kampot Province, Over 25 yr-olds)*.

#### Missed distribution opportunities

Finally, in three focus groups, women missed the household distribution opportunity and were not sure how to get in contact with the program in order to get their voucher.

They came and tried to find [her] at home, but she was not home. They went past [her] house and asked everyone for her name. So, now she does not have the voucher (Kampot Province, 18–25 yr-olds).

### Sub-theme 3: redemption

#### Fear of hidden costs

Even with the voucher, in two our of nine focus groups women still felt that they would be expected to pay their midwife “tea money” for delivery services. The voucher management agency explicitly prohibits this practice, but thus far, had not been able to enforce this restriction. The fear of having to pay these additional fees deterred them from using the voucher for delivery.

#### Limited locations and services

In addition, women in three groups reported that they had wanted to use their vouchers at non-accredited health facilities or for non-covered services at accredited facilities and were turned down.

The bad point is that we cannot use it at the private clinic. They don’t accept it. We can only use it at the public clinics (Kampot Province, Over 25 yr-olds).

#### Confusion with other programs

There have been several other social protection programs implemented in this area, including the Health Equity Fund that allows women to receive free delivery services at the government hospital and the Marie Stopes International voucher program that offered vouchers to poor households for reproductive health services for short periods of time. In three out of nine focus groups, when women were asked to show their vouchers, they were not always sure which one to present.

Is it the blue voucher? The small one? I didn’t bring it with me. I mixed it up (Kampong Thom Province, Over 25 yr-olds).

#### Negative reactions from providers at hospitals

In three of the nine focus groups, women reported that when they presented at the provincial hospital with a voucher, they faced discriminatory treatment because they were not paying with money. There were only a few reported cases of discrimination and it was unclear which facilities these women had gone to and which subsidy program they were using (RH Vouchers, Health Equity Fund, Marie Stopes Program). It is also unclear if the negative reactions were a result of misunderstandings around program logistics, but this topic had great significance to the women who reported it during the group conversation.

When we went to the [provincial hospital] and showed them the poor voucher and told them that we are poor, they said, ‘I don’t eat that paper, I eat with money’ (Kampot Province, Over 25).

The voucher holder is not treated well. [The providers at the provincial hospital] are just relaxed and let us stay without paying attention. They do not come often to see us like the people who pay. We have to give them some tea money for them to take care of us (Kampong Thom Province, Over 25 yr-olds).

A handful of smaller issues came up in conversations, but were not classified as themes, including a couple of women who reported that they forgot to bring their voucher to their clinic visit and several women who thought that because the services were free, they may not be of high quality. These inconveniences and negative views noted by a smaller number may also inhibit voucher use.

## Discussion

This study explored the experiences of women accessing reproductive health services in rural Cambodia with and without a voucher. Overall, women expressed positive feelings towards the voucher program, reporting that they sought earlier and more frequent care with the vouchers. In addition, women described a feeling of security and mental ease knowing that they had access to free care and a transportation stipend. Women also gave descriptive feedback about their experiences seeking care prior to the voucher program and the results of these discussions offer a number of considerations for voucher program design and management that could enable managers to better tailor the program to the communities they are serving.

Increasing the utilization of services is one of the most important aims of voucher programs. Before the voucher program existed, utilization patterns were impacted by a number of factors that ranged from physical access to clinics to experiences with side effects of contraception and relationships with providers. Women described a range of experiences when negotiating birth spacing and limiting with their partners, getting support from health care providers, and trying out various types of contraception. For example, women said that they did not feel comfortable starting conversations with their husbands about using contraception until they had given him three or four children. Women stated that health care providers did not always give them the space and time to discuss types of contraception and possible side effects. And many women experienced negative side effects such as irregular bleeding, weakness or discomfort, which resulted in their discontinuation of that method – or all methods in some cases. All of these experiences affect women’s baseline utilization of health care services and any program that aims to increase utilization of these services should take into account the information and sensitization needs of a community before implementing the program.

This study attempts to bring in women’s voices early enough during program roll-out that beneficiaries can have input on what is working and what could be improved. Taking into account women’s existing perceptions and experiences with family planning may inform distributors’ explanation of the program benefits and the importance of counseling around these services. In addition, providers at accredited facilities may be able to pro-actively address some of the negative impressions of certain contraceptives and to talk to women about how to begin a dialogue with spouses about the benefits of well-spaced births.

### Implications for future voucher programs

Findings from this exploratory study can offer the following recommendations for program improvement. We recommend:

•Implementation of a more transparent poverty grading process

•Delivery of clear information about where to obtain a voucher if a house is missed

•Dissemination of contact information for an independent agent apart from village chiefs

•Improved training of distributors not only on program logistics, but on community-specific topics such as family planning decision-making

•A better branding strategy for easy voucher identification

•Explicit provider guidelines and information to patients about unofficial payments

•A strategy for accepting anonymous complaints when women have negative experiences using their voucher

The distribution of the vouchers to individual households was seen as a successful strategy for encouraging poor families to use reproductive health services as compared to before the voucher program. But a more transparent poverty grading process that takes into account individual poverty in a more comprehensive way may be necessary with particular strategies in place for community input. In addition, if a house was missed during the distribution, clear information about where to obtain a voucher should be disseminated. Program participants should be able to contact an independent agent in order to obtain a voucher – this system should not rely on the village chief, although the chief could be an important local resource and should receive adequate education on program logistics and purposes. In addition, women did not discuss the counseling or health information that should have been delivered by distributors. Particularly when such strong feelings about side effects were expressed, there is a need for additional training of distributors not only on program logistics, but on community-specific topics that should be included in counseling, such as what side effects to expect and how to initiate a conversation about family planning within a household. Finally, a better branding strategy that would help eligible households be able to identify this particular program from the other social protection measures that are in place in Cambodia may improve usage.

Many women that participated in these focus groups have not yet had the chance to redeem their vouchers because they did not yet need them or were waiting to see the early adopters experiences. When anticipating using them, many women expressed some confusion about how and where to use the voucher, if there will be additional charges, and how providers will receive women presenting with vouchers. As a result, accredited providers should also be counseled on how the program works and how they can make poor women feel welcome in their facilities. In addition, more explicit provider guidelines and information to patients about unofficial payments to midwives may need to be established and disseminated to both providers and patients. Current voucher programs may not be able to eliminate these unofficial payments and new ways to manage tea money transactions need to be explored such as provider incentives. There may also be the need for a way to anonymously issue complaints when women have negative experiences using their voucher, although this many not be an acceptable practice within the community. Quality issues clearly need to be followed up by some remediation by the VMA as otherwise the early negative experiences may contribute to a lack of wide acceptance by the population in greatest need for such program interventions.

### Limitations

There are several limitations in conducting this research. First, because of our recruitment strategy, we are not able to ascertain information about those who did not choose to participate, which is particularly important given the role of the village chiefs. Traditionally, we would not rely upon a potentially biasing source for recruitment, but at the same time, without the support of the chief, the study in this context would not have occurred. Thus, while there may be some disadvantages to our recruitment strategy, without this entry, we would not have been able to capture any of these women’s experiences.

Second, there was variability between facilitators in terms of their ability to encourage interaction and discussion between participants. Fortunately, an examination of participant responses by facilitator revealed that although some facilitators encouraged more discussion, the content of the responses did not vary greatly. However, across all groups, young women spoke less overall than older women and none of the facilitators were successful in getting younger women to speak more. An effort was made to examine and integrate the content from the younger women’s group despite this limitation. An implication of this finding is to conduct focus groups in the future that are specifically designed to provide the most comfortable environment for younger women. Third, there was variability in the transcription and translation process. Although the authors trained all transcriber and translators using the same training manual and asked them to use the same standardized transcription guide, it is still possible that important responses were misinterpreted or that meaning was lost in translation. Translators read and commented on an early report of study findings with an eye towards misinterpretation. For the few issues that came up, the author went back to the facilitators for clarification and several corrections were made based on this feedback.

Fourth, focus groups were held at village chiefs’ houses in an open-air setting and because most women who attended were familiar with each other, there is concern about the degree of privacy that participants experienced and about the impact of the lack of privacy on their willingness to disclose feelings about personal topics such as those related to reproductive health, especially family planning requiring husband’s consensus. After three focus groups (one with older women and two with younger women), Cambodian research assistants quietly asked several women from each group if they felt comfortable speaking freely about their healthcare behaviors in this environment and all women who were asked said they did. This issue may still have impacted the results of this study, but the candid responses to issues such as “tea money”, the expressions regarding how women felt that they were treated when they had vouchers, and the need to pursue family planning, even if their husbands were not supportive, provides some sense that women did feel comfortable in disclosing personal information in a public venue. Unfortunately, the authors were not able to conduct individual interviews during this phase of the project.

Finally, researchers who were not from the same community as the study participants conducted the data analysis and this data was interpreted from their cultural perspective. The authors asked their Cambodian research assistants, who attended all focus groups and participated in note taking, memos and debriefing to review the results of the data analysis and their feedback was fully integrated particularly related to discussion around traditional services and tea money.

## Conclusion

Early information from beneficiaries who are interacting with a new program can lead to timely and responsive changes in program activities that can help to maximize program success. This methodology produced findings relevant for program planning which are more useful that estimated responses based on hypothetical scenarios, which have not shown to produce accurate results. Voucher programs are still considered a new strategy for increasing the utilization of health services for a targeted population. The evidence for the effectiveness of voucher programs is still being developed. In order to encourage utilization through these programs, early beneficiary feedback should be incorporated into program design. In addition, comparing the voucher strategy to other demand-creation or subsidy programs within this context, in terms of cost effectiveness and quality, would be an important next step. This study highlights the importance of tailoring voucher programs to community needs, a strategy that can lead to more successful program adoption and outcomes.

## Abbreviations

VMA: Voucher management agency; TBA: Traditional birth attendant

## Competing interest

The authors declare that they have no competing interests.

## Authors’ contributions

CB conceptualized the study, managed the data collection and analysis, and took the lead on drafting the manuscript. JF contributed to study design, data collection and analysis and drafting the manuscript. CB contributed to the study design and reviewed the manuscript. BB contributed to study design, data collection, and reviewing the manuscript. All authors read and approved the final manuscript.

## Pre-publication history

The pre-publication history for this paper can be accessed here:

http://www.biomedcentral.com/1472-698X/13/13/prepub
